# Quality and learning aspects of the first 9000 spirometries of the LifeGene study

**DOI:** 10.1038/s41533-018-0073-y

**Published:** 2018-02-19

**Authors:** Mikaela Qvarfordt, Martin Anderson, Magnus Svartengren

**Affiliations:** 1 Department of Laboratory Medicine, Division of clinical physiology, Karolinska Universitetssjukhuset, Stockholm, Sweden; 2 Department and Medical Sciences, Division of Occupational and Environmental Medicine, Uppsala University, Uppsala, Sweden

## Abstract

Spirometry requires the patient to cooperate and do the manoeuvre 'right' for reliable results. Algorithms to assess test quality as well as educational recommendations for personnel are defined in guidelines. We compared the quality of forced spirometry tests performed by spirometry technicians with little or no previous experience of spirometry using spirometry systems with different modes of feedback. In both cases, the spirometry technician received general feedback on the screen based on ATS/ERS guidelines, such as 'exhale faster' and 'exhale longer'. The major difference was whether quality grading system of the complete session was available simultaneously on screen, or in the printed report afterwards. Two parts of the same population-based study (LifeGene), the pilot (LG1) and the first part (LG2) of the subsequent study, were compared retrospectively. In LG1 (on-screen grading) approved examination quality was achieved for 88% of the 10 first subjects for each spirometry technician compared to 70% in LG2 (printed grading afterwards). The corresponding values after 40 subjects was 94 % in LG1, compared to 73% in LG2, and after the first ten subjects there was no apparent quality improvement in either LG1 or LG2. The quality for LG1 is among the highest reported in the literature even though the spirometry technician were relatively inexperienced. We conclude that on-screen grading in addition to general technical quality feedback is powerful in enhancing the spirometry test session quality.

## Chronic lung disease: On-screen feedback for effective spirometry testing

On-screen, real-time feedback for technicians and patients during spirometry raises the efficiency and quality of tests. Spirometry measures air flow passing through the lungs to determine lung function. However, the test relies on patients co-operating and performing at their best to achieve high quality results and avoid mis-diagnosis. As part of the LifeGene study at Karolinska University in Stockholm, Sweden, Mikaela Qvarfordt and co-workers trialled an automated method of providing feedback during testing to inexperienced spirometry technicians and their patients. 9000 patients were split into two groups, LG1 and LG2. LG1 received on-screen feedback plus a quality grade during testing, while LG2 received grading feedback after tests had finished. For every technician, approved quality tests were achieved for 94 per cent of their first 40 patients in LG1, compared with 73 per cent in LG2.

## Introduction

Forced spirometry is widely used as the major tool to diagnose chronic obstructive pulmonary disease (COPD), which is about to be become the third most common cause of death,^1^ making spirometry of crucial importance in a public health perspective. An aggravating factor is that spirometry tests require the patient not only to cooperate, but also to perform maximally to give reliable results of good quality.^2–6^ How to ensure spirometry quality, and how poor quality may affect the diagnosis, have been studied in several previous research projects with different approaches,^2,5,7–16^ all in pursuit of the same goal; reliable and cost-effective testing.

The concept of high quality can have different meanings. While quality depends on initial training and education of the spirometry technician (from here called technician), the ability to maintain quality over time is also of importance. Supervised regular assessment have been studied to prevent quality decrease over time after initial training or study onset.^5,14,17^ For example, Burgos et al.^18^ used internet based support for a group of test centres, and found better quality when compared to the control group of centres. Others^19^ have pointed out the importance of visual inspection of spirometry curves. These studies have mostly studied effects of feedback on test session after the test is completed, opposed to the present study investigating automated quality feedback during testing.

LifeGene is a population-based study, with Karolinska Institutet in Stockholm as host (www.lifegene.se), in which spirometry is included in the physiological testing of participants. The actual LifeGene study (called LG2) was preceded by a massive pilot study (called LG1). A standardized grading system^10^ consisting of five defined quality grades is used to assess spirometry quality, where a high quality grade means high degree of repeatability, interpreted as maximum effort. The quality grade thereby reflects the quality of the whole test session.

Two different office spirometers (and consequently two different software) were used, one in LG1 and another in LG2, both presenting feedback on individual tests (exhalations) on-screen, but differed in the way they presented the quality grade of the spirometry session. This difference between LG1 and LG2 gave us the opportunity to compare tests with automated real-time quality feedback given as quality grade during the test session (LG1), with tests where the same feedback was given directly after the session (LG2).

The purpose of this study was to investigate whether spirometry technicians after a relatively short training could perform spirometry tests to an approved quality and to maintain it over time as well as to compare the two parts of the study to investigate the value of the on-screen quality grading itself.

## Results

### Summary of participant data LG1 and LG2

A total of 5043 people participated in LG1, with an age range between 6 and 74 years. During the first six months, 4379 people participated in LG2, with an age range between 10 and 80 years. Since invitations were directed to persons 18-45 years, the number of participants with age >45 years was relatively small; in LG1 228 of 5043 (4.5%) and in LG2 944 of 4379 (21%). 79 subjects were <18 years of age in LG1 and 55 in LG2. Demographic data are presented in Table 1.

**Table 1:** Demographic and spirometry data of participants LG1 and LG2

		Age, mean (SD)	Height, mean (SD)	Weight, mean (SD)	FVC, mean (SD)	FVC % pred,^a^ mean (SD)	FEV1, mean (SD)	FEV1 % pred,^a^ mean (SD)
*LG1*	Female	32.9 (8.44)	167.6 (6.33)	66.3 (11.34)	4.25 (0.59)	107,1 (13,0)	3.47 (0.49)	104.3 (12.6)
	*n*=3015							
	Male	33.8 (8.36)	181.1 (6.56)	83.0 (12.54)	5.86 (0.81)	105,2 (11,8)	4.67 (0.66)	102.9 (11.8)
	*n*=2028							
*LG2*	Female	36.9 (13.45)	167.0 (6.38)	65.8 (10.41)	3.93 (0.68)	110,4 (14.0)	3.21 (0.55)	107.5 (13.2)
	*n*=2715							
	Male	36.8 (12.67)	181.0 (6.74)	82.3 (11.73)	5.60 (0.95)	110,8 (14.8)	4.42 (0.73)	106.8 (13.6)
	*n*=1664							

^a^Predicted values according to GLI12^20^

### Spirometry data

FVC and FEV1 for all the included participants in LG1 and LG2, with at least one approved spirometry manoeuvre are presented in Table 2, and compared to GLI reference values.^20^

**Table 2:** Grading of quality in terms of repeatability; LG1 (Jaeger) vs. LG2 (MIR)

Software grading by Jaeger used in LG1	Software grading by MIR used in LG2		
Grade	Definition	Grade	Definition
1	At least two approved tests. Difference in both FEV1 and FVC of <100 mL	A	At least two acceptable manoeuvres, with the highest two FEV1 values matching to within 100 mL and the largest two FEV6 values within 100 mL
2	At least two approved tests. Difference in both FEV1 and FVC of 101–150 mL	B	At least two acceptable manoeuvres, with the FEV1 values matching to within 101 to 150 mL
3	At least two approved tests. Difference in both FEV1 and FVC of 151–200 mL	C	At least two acceptable manoeuvres, with FEV1 values matching to within 151 to 200 mL
4	At least two approved tests. Difference in both FEV1 and FVC of>201 mL	D	Only one acceptable manoeuvre, or more than one, but the FEV1 values not matching to within 200 mL (with no interpretation).
5	No approved tests	F	No acceptable manoeuvres (with no interpretation)

### Examination quality

**Fig. 1:**
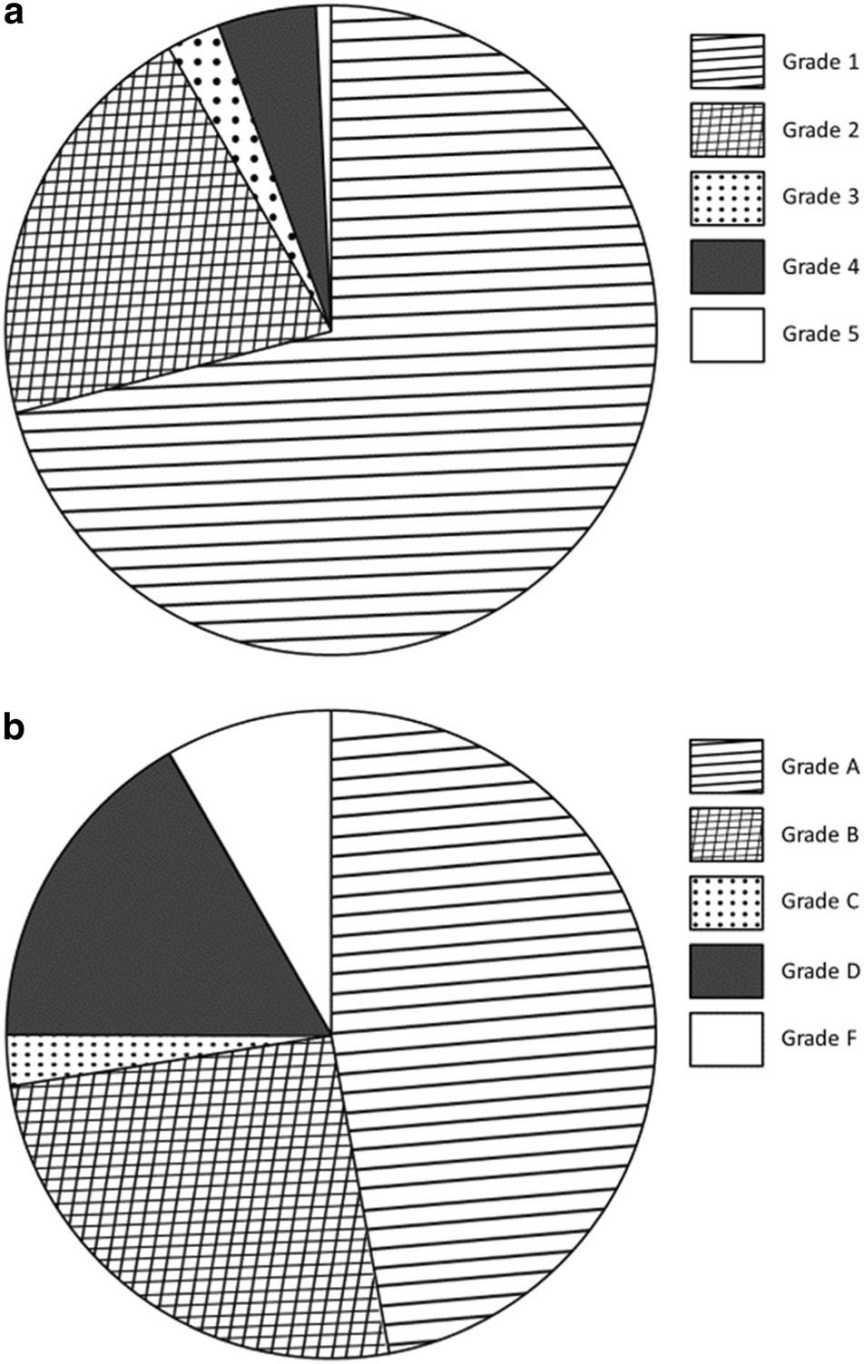
Quality grade for all subjects in LG1 and LG2 showing higher grades (i.e., better quality) with continuously displayed on-screen quality grade in LG1 compared to LG2 when quality grade was obtained after the test was completed

**Fig. 2:**
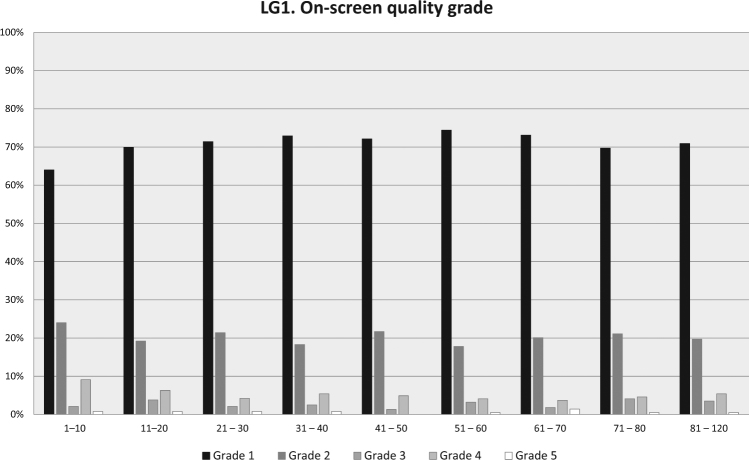
LG1—quality grade displayed continuously on screen, Subjects in groups arranged in consecutive order

**Fig. 3:**
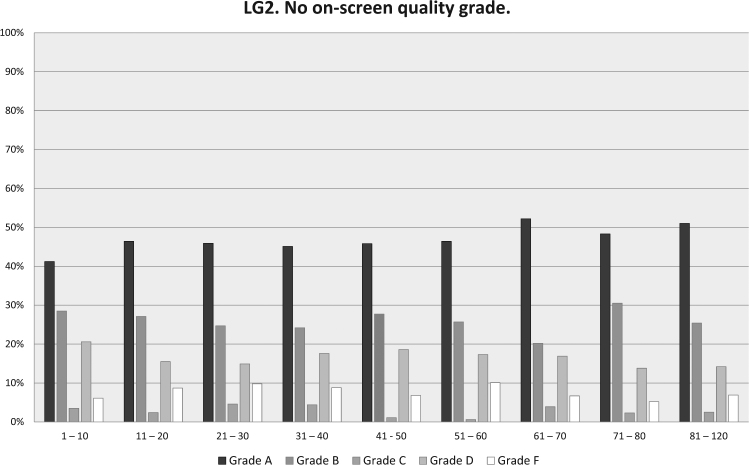
LG2 quality grade not visible on screen, only in rapport after completed test. Subjects in groups arranged in consecutive order

In LG1, with on-screen grading, 92% of all tests were within 150 mL (grade 1 + 2). In LG2, with grading afterwards, only 73 % of tests were within 150 mL difference (grade A + B) (Fig. 1). In LG1 88 % of the technician’s first 10 subjects reached ATS / ERS standard compared to 70 % in the LG2. After 40 subjects 94 % reached approved level in LG1, 73% in LG2 (Figs. 2 and 3). It also should be noted that in LG1, only 1% of the subjects were not able to complete a single approved attempt, unlike LG2, where the corresponding figure was 8%. The fraction of subjects >45 years of age was higher in LG2, but this did not cause the difference. For subjects over 45 years, grade 1 + 2 was 92% in LG1 and 81% in LG2. For subjects under 45 years, grade 1 + 2 was 92% in LG1 and 70% in LG2.

When measuring quality over time for the whole test centre, the steep learning curve was over after 6 weeks and no significant changes occurred after that (data not shown).

## Discussion

For research purposes, forced spirometry was conducted in a general population in the pilot (LG1) and the first six months (LG2) of the LifeGene study. All tests were done at a single test centre, which, to our knowledge, is quite rare in these types of studies. The main objective in the present study was to assess the influence of simultaneous automated on-screen quality grading, compared to grading given in the report after the session (which was the major difference between LG1 and LG2), and to investigate quality development during the study.

A large fraction of the spirometry tests conducted in both LG1 and LG2 met the ATS and ERS standards of quality and reproducibility. However, there was also a considerable difference in quality between the exceptional results in LG1 compared to the less good results of LG2. The difference was seen from the first ten subjects tested by each technician and throughout the study.

In general, feedback on quality will improve quality. There is no doubt that monitoring spirometry results and giving technicians personal feedback, visual inspection by experts, telemedicine support etc, can all be beneficial. However, for large scale studies, one question will be how to achieve high quality in the most efficient way. While other investigators^4,5,11–13,18^ have shown good results using feedback on completed tests, the present study shows that if used with relevant instructions, automated real-time feedback of quality grade can also have a significant impact on quality, altering what happens during the session—which of course is very efficient.

In the Bold study that included 9893 participants with test centres in 14 countries, approximately 90 % of the tests had a reproducibility of 150 mL or less.^21^ Some previous studies have set the limit for approved quality as an allowed difference between best and second-best exhalations (for FEV1 and FVC) to more than the 150 mL corresponding to Grade 2/B or better. This makes numerical comparisons with our study somewhat difficult. For example, the World Trade Centre Responder program in New York City, investigated 13,599 volunteers and ~80% achieved a repeatability of up to 200 mL,^11^ to be compared to LG1, where 92%, and LG2 73% of the subjects reached a reproducibility of less than 150 mL. What differentiate study design in these studies in relation to LifeGene are several things: In LifeGene all spirometry tests were performed at a single centre as opposed to a data collection between both different test centres and sometimes even different countries. The training period in LifeGene was less extensive than both The Bold study^21^ and the WTC study.^11^ Further, the technicians’ individual session quality was monitored in both Bold and the WTC: If the quality fell below a certain level, extra training was initiated before the technician were allowed to continue working. In LifeGene there was no similar monitoring and the 1-day training was only followed by a 'follow-up' meeting a month after study start. This meeting was aiming for a discussion of examinations, and answering questions, but no individual quality check was done prior or during this meeting.

The real-time feedback in LG1 may have led to a feeling of competition in game-like way, in turn leading to more trials (exhalations) being done when the technician was striving for a better result. There is no indication that test sessions LG1 took longer time than in LG2, but unfortunately, we don´t have data on number of trials in each session.

The two groups (LG1 and LG2) of subjects were examined under almost exactly the same circumstances and study plan, except for the spirometers and their software. Both spirometers were approved as diagnostic devices by ATS standards, specified to differ not more than ±2%. We consider the different hardware unlikely to cause the differences we see in quality outcome. The difference in quality grade between LG1 and LG2 could not be attributed to the minor discrepancies between the two grading systems (1–5 and A–F), since the software used in LG1 used more extensive criteria and still gave higher results.

When LG2 began, the advertising was intensified leading to the higher proportion of subjects over 45 years in LG2. However, the subjects >45 years actually showed higher quality, so this cannot explain the lower quality in LG2.

Other possible reasons for the difference in quality could of course be the selection of technicians. The same staff was in charge of recruitment, but the time frame differed about a year. When compared, the recruited persons overall appears to have been similar. A few of the technicians who worked in LG1 continued their employment in LG2. This means that there was a few more technicians with training and experience of spirometry in LG2. If this had any impact on quality it would have been to increase it in LG2 (which showed lower quality). Also for the participants the recruitment process was the same but about a year apart, and we haven’t found any reason to believe that this affected the results significantly.

Improvement of quality was detected during the first 40 participants, but after that there was no trend of improvement, not even in LG2 that showed the lower quality. The increase in quality was surprisingly fast and somewhat contradicts recommendations made by ERS, ATS and other professional organizations, as well as previous studies on education and training required for good quality. We can only speculate in the reasons for this finding of rapid learning: In our study the technicians all worked at the same test centre, which we believe was beneficial for the learning process due to the internal and informal exchange of knowledge and practices, but we have no actual data to support this. However, even if technicians are spread geographically, they normally work with colleagues in a primary health care centre or similar, or perhaps as in the study by Burgos et al.,^20^ have the possibility to share knowledge on internet discussion forums. The contribution of informal groups on individual learning is probably significant, but we have found no studies on this matter in the field of spirometry. In the present study, the frequency of tests was high (up to seven participants per technician and day). Optimum frequency is unknown, but the high number of sessions per technician and day probably contributed significantly to the fast learning process.

This study shows that an exceptional high degree of acceptable tests can be achieved within weeks in in large population-based studies with very little initial training, as in LG1. This enables monitoring systems to judge the overall quality of large scale studies, or screening projects, relatively soon after the start. Our conclusion is the fast learning curve primarily is the result of intense testing, and we also speculate that a group learning effect had a significant impact. Finally, from the comparison between LG1 and LG2, we conclude that simultaneous quality grading (in addition to standard acceptance criteria) have a major impact on examination quality, given it is configured as simultaneous on-screen feedback and used in real-time during testing.

## Material and methods

Informed consent was obtained for the participants of the LifeGene project and thereby an agreement to use collected data in research projects. In addition, the present study was approved by the Regional Ethical Review Board (Etikprövningsnämnden) in Stockholm. LifeGene invited index persons between 18 and 45 years of age randomly from the government person address register (Statenspersonadressregister, SPAR), which includes all persons who are registered as residents in Sweden. The invitations were sent by mail and written in Swedish. The index persons were encouraged to invite family members to the study (including persons under 18, or over 45 yrs). There was also a possibility for anyone to register for participation at the LifeGene web site, and this was advertised in newspapers. The study was scheduled to examine 500,000 volunteers over several years. The pilot study (LG1), involved 5000 subjects. After LG1 was finished there was a 18 month evaluation period, after which the study continued (LG2). Both LG1 and LG2 included a questionnaire and a visit to the test centre. Each participant had an effective time at the centre of about 45 min with a technician in a designated room where a number of tests where made: blood sampling, measurements of length, weight, circumference measures of thorax, waist and hips, bio impedance, audiometry, and spirometry. The spirometry was performed according to ERS/ATS guidelines.^6^ The main spirometry parameters obtained where forced vital capacity (FVC) and forced expiratory volume first second (FEV1), as well as the ratio between FEV1 and FVC. The quality grading was used as an indicator of examination quality and was compared retrospectively between LG1 and LG2. The study was conducted according to guidelines published by STROBE Statement.^22^

### Equipment/Data System

A computerized process support for the entire visit was constructed, in order to simplify, streamline and ensure proper data collection. One of the modules in this process sequence led directly to spirometry programme (LG1: Lab Manager, Jaeger, Höchberg, Germany; LG2: WinSpiro, MIR, Rome, Italy). All data, including data on the test quality, was exported to a central database. The seven spirometers used in LG1 (Jaeger Masterscreen, Jaeger, Höchberg, Germany) were all calibrated daily. A bacterial filter was used for each participant, and cleaning of the spirometers was done at the end of the day. The flow sensor being used in LG2 (FlowMIR, MIR, Rome, Italy) was for single patient use only (hence, used without filter), and was delivered calibrated from the manufacturer ready to use. Prior to LG2 a 3 L calibration syringe (Sensormedics, San Diego, CA, USA) was used to verify that the two spirometers showed expected volumes ±2%.

### Quality Feedback

Both software programs were adapted so that only the following parameters were displayed on the screen: FEV1, FVC, PEF and the ratio between FEV1 and FVC. Both programs use ERS/ATS criteria to verify that the start of expiration is fast enough and without hesitation, and that the ending is long enough and not aborted too early. Overall, this indicates that the participant performed each test (exhalation) correctly. All technicians were instructed to follow potential instructions on screen concerning back-extrapolated volume, end expiratory flows, and exhalation time. Number of attempts was set to a minimum of three and maximum eight. Both programs also checked that the different approved tests (expirations) did not differ too much from each other, using criteria for reproducibility called 'office grade'. The concept of office grade was explained to all technicians. Approved examination quality according to the ATS / ERS standards is equivalent to Grade 1 or 2 / A or B. Grading criteria for the two spirometers are given in Table 2.

#### The main difference between LG1 and LG2

In LG1 technicians had continuously updated information of office grade on screen during the whole session, (which typically improves during the session) and were urged to actively work towards 'Office-grade 1' (corresponding to office grade A). In LG2 technicians had access to the office grade for the session only afterwards, when it was printed on the report.

### Spirometry technicians

The technicians performing the testing consisted of both what in Sweden translates to Biomedical Scientists (internationally also known as Medical Laboratory Technologist) as well as nurses, in about equal proportions. They were recruited mostly through advertisements in local newspapers. Most of them had no previous experience of spirometry at all, and the rest very little. Both LG1 and LG2 used the same training arrangements: The technicians were given a 1-day training course in groups of ten, including lectures and hands-on training. A few days before the start of the study additional hands-on training were given at the test centre during a half-day session.

### Statistic calculations

Calculations were based on spirometry data on all cases >18 years old (except for the three subjects showing implausible values due to typing errors) reported to the central storage system. This means that also data tagged 'Grade 5' or 'Grade F' (meaning that at least one attempt was made, but no acceptable tests were registered) were included. From these data descriptive statistics were analysed. All analyses were performed using the software SPSS version 22 (IBM, Chicago, IL, USA) on a personal computer with Windows 7 platform. For GLI reference equations the *GLI-2012 Excel Sheet Calculator* by Sanja Stanojevic (Version 4, 25 May 2014) was used (downloaded from www.ers-education.org).

### Data availability

All calculations were based on data extracted from the Life Gene database. Info on applications for data from LifeGene can be made at https://www.lifegene.se/For-scientists/.

## References

[1] WHO. World Health Statistics 2008. *World Health Organization*. (Part 1), http://www.who.int/gho/publications/world_health_statistics/EN_WHS08_Full.pdf?ua=1 (2008).

[2] Hegewald MJ, Gallo HM, Wilson EL (2016). Accuracy and quality of spirometry in primary care offices. Ann. Am. Thorac. Soc..

[3] Enright PL (2003). How to make sure your spirometry tests are of good quality. Respir. Care.

[4] Tan WC (2014). Quality assurance of spirometry in a population-based study - predictors of good outcome in spirometry testing. COPD J. Chronic Obstr. Pulm. Dis..

[5] Enright PL, Johnson LR, Connett JE, Voelker H, Buist AS (1991). Spirometry in the Lung Health Study: 1. methods and quality control. Am. Rev. Respir. Dis..

[6] Miller MR (2005). Standardisation of spirometry. Eur. Respir. J..

[7] Burgos F (2014). Clinical decision support system to enhance quality control of spirometry using information and communication technologies. JMIR Med. Inform..

[8] Marina N (2016). Economic assessment and budgetary impact of a telemedicine procedure and spirometry quality control in the primary care setting. Arch. Bronconeumol..

[9] Banks DE, Wang ML, McCabe L, Billie M, Hankinson J (1996). Improvement in lung function measurements using a flow spirometer that emphasizes computer assessment of test quality. J. Occup. Env. Med..

[10] Ferguson G, Enright P, Buist S, Higgins M (2000). Office spirometry for lung health assessment in adults* a consensus statement from the national lung health education program. Chest.

[11] Enright PL, Skloot GS, Cox-Ganser JM, Udasin IG, Herbert R (2010). Quality of spirometry performed by 13,599 participants in the World Trade Center Worker and Volunteer Medical Screening Program. Respir. Care.

[12] Enright PL (2011). Quality of Spirometry tests performed by 9893 adults in 14 countries: The BOLD Study. Respir. Med..

[13] Enright PL, Beck KC, Sherrill DL (2004). Repeatability of spirometry in 18,000 adult patients. Am. J. Respir. Crit. Care Med..

[14] Eaton T (1999). Spirometry in Primary Care Practice: The Importance of Quality Assurance and the Impact of Spirometry Workshops. Chest.

[15] Licskai CJ, Sands TW, Paolatto L, Nicoletti I, Ferrone M (2012). Spirometry in primary care: An analysis of spirometry test quality in a regional primary care asthma program. Can. Respir. J..

[16] Kunzli N (1995). Variability of FVC and FEV1 due to technician, team, device and subject in an eight centre study: Three quality control studies in SAPALDIA. Eur. Respir. J..

[17] Enright PL, Connett JE, Kanner RE, Johnson LR, Lee WW (1995). Spirometry in the Lung Health Study: 2. Determinants of Short-Term Intraindividual Variability. Am. J. Respir. Crit. Care Med..

[18] Burgos F (2012). Telemedicine enhances quality of forced spirometry in primary care. Eur. Respir. J..

[19] Müller-Brandes U (2014). LUNOKID: can numeric ATS/ERS quality criteria replace visual inspection of spirometry?. Eur. Respir. J..

[20] Quanjer PH (2012). Multi-ethnic reference values for spirometry for the 3–95 year age range: the global lung function 2012 equations. Eur. Respir. J..

[21] Buist AS (2005). The Burden of Obstructive Lung Disease Initiative (BOLD): rationale and design. COPD.

[22] von Elm E (2007). STROBE Initiative. The Strengthening the Reporting of Observational Studies in Epidemiology (STROBE) statement: guidelines for reporting observational studies. J. Clin. Epidemiol..

